# The evaluation of a healthcare passport to improve quality of care and communication for people living with dementia (EQuIP): a protocol paper for a qualitative, longitudinal study

**DOI:** 10.1186/s12913-016-1617-x

**Published:** 2016-08-09

**Authors:** Gerard Leavey, Aine Abbott, Max Watson, Stephen Todd, Vivien Coates, Sonja McIlfactrick, Brendan McCormack, Bethany Waterhouse-Bradley, Emma Curran

**Affiliations:** 1 The Bamford Centre for Mental Health & Wellbeing, Ulster University, Cromore Road, Coleraine, Northern Ireland; 2 Royal College of Practitioners, Belfast, Northern Ireland; 3 Queen Margaret University, Edinburgh, Scotland; 4 Western Health and Social Care Trust, Derry-Londonderry, Northern Ireland; 5 Northern Ireland Hospice, Belfast, Northern Ireland

**Keywords:** Dementia, Self-management, Personhood, Doctor patient communication, Qualitative longitudinal research, Realist review, Study protocol

## Abstract

**Background:**

There is an urgent need for the development of simple communication tools that convey the strengths, assets, and healthcare needs of people living with dementia. A Healthcare Passport may improve communication with range of health and social support services, enhancing quality and continuity of care, and to permit a consideration of the challenges and how these might be managed effectively and compassionately. This study aims to evaluate the acceptability and use of this type of intervention for people living with dementia and their carers.

**Methods/Design:**

This is a qualitative longitudinal study informed by a critical realist review. The participants will be individuals identified as having mild-moderate dementia and informal carers. The in-depth interviews will occur at three points over the course of 18 months as they use the passport. This will be supplemented by analysis of the content of the passports and information from health and social care providers on the daily practicalities of using the passport in a range of healthcare settings.

**Discussion:**

By using a critical realist review and a qualitative, longitudinal approach, the study allows for the assessment of a complex intervention in a manner which goes beyond evaluating the basic efficacy of the passport, but looking more deeply at how it worked, for whom, and in what context. It has the potential to develop new data on how interventions improve communication across a range of service providers, while encouraging health and social care professionals to respect and encourage the development of self-management and retention of personhood throughout the progression of life-limiting illnesses.

## Background

Improving the quality of life for people living with dementia should be underpinned by the communication of their needs, strengths and life situation. Unfortunately, such communication remains poor. Moreover, shared decision-making and planned care is limited, and services still neglect the personhood of the individual, eschewing the personal, spiritual and social characteristics of significance from treatment and care. Simple communication tools are needed in order to facilitate self-management, and provide a mechanism through which individuals may indicate future care preferences.

### Barriers to holistic care in dementia

One third of the current population, aged over 65, will live out their lives with dementia. These numbers will double by 2040, accompanied by a tripling of dementia care costs [1, 2]. We require imaginative ways of living with, and managing, dementia care so that people with dementia can lead full and meaningful lives. Ideally, people living with dementia should determine the appropriate services and when and how these are provided. Currently, this is not the case for many people [3]. When dementia is accompanied by multiple and complex health problems, care and treatment is often experienced as disjointed and compartmentalized, and lacking consultation [4–6].

Even the best services may be unintentionally complicit in the disablement and marginalization that often accompanies dementia [7]. In this context, medical care can be regarded as mechanistic and lacking compassion [8]. Person-centred care considers the whole person, accommodating each individual's qualities, abilities, interests, preferences and needs [9]. Families may ensure that these aspects are acknowledged and upheld. However, the range of informal caring [10, 11], may be taken for granted by professionals. Evidence from our consultation with carers and supported by research evidence reveals a weary resignation of explanation and negotiation with an ever-changing cast of professionals. This is likely to be compounded by challenges to knowledge and help-seeking in dementia care, difficulties of communicating need and decision-making [12, 13].

The evidence on interventions designed to support individuals with dementia and families who act as care partners is unclear [14, 15]. One systematic review [16] suggests that individualized interventions that use problem solving and behaviour management offer the best evidence of effectiveness. However, few such interventions exist to assist integrated care delivery and these lack good evidence on their effectiveness. A person-centred approach to psycho-social support of family carers demands a more nuanced understanding of the unique challenges experienced in individual caring relationships. Evidence supports the use of well-designed psycho-educational or multi-component interventions for caregivers of people with dementia [17]. The factors that appear to positively contribute to effective interventions are those which: provide opportunities within the intervention for the person with dementia as well as the caregiver to be involved; encourage active participation by caregivers; offer individualized programs and provide information on an ongoing basis, with specific information about services and coaching regarding their new role [18].

### Improving communication through a healthcare passport

The Northern Ireland Public Health Agency in partnership with the Royal College of General Practitioners and a consortium of voluntary sector organisations and service users, helped develop a Healthcare Passport, designed to assist communication for people with long-term health problems. The passport is an expandable document in which key personal, social care and clinical information is recorded (by, or on behalf of, those living with dementia). It is constructed to allow a compendium of information about the person with dementia such as their close family members and friends, activities, interests, and beliefs. It will also provide brief but key information about dementia. Other sections are available to encourage the entry of information from health and social care professionals, including information on co-morbid diagnoses, medication and other treatments. It is designed to allow continuous updating by family and health professionals, acting as a ‘live’ synchronized record of changing need, service contact and information provision. Importantly, the passport may, additionally, stimulate among health and care professionals a sense of the personhood of the individual with dementia.

Our aims in this study are two-fold. First, to contribute to the evidence base on the use of services, communication needs and decision-making processes of people with dementia and their family caregivers as these unfold over time. Secondly, to clarify the acceptability and usage of communication tools for people living with dementia and how such tools may be improved. Additionally, we seek to apprehend any implementation issues in preparation for a randomised controlled trial of this complex intervention.

We will address the following questions:

1. Is a Healthcare passport acceptable and useable for people with dementia, their family caregivers and health professionals?
2. Is a sense of ‘personhood’ in the midst of dementia conveyed by the use of Healthcare passport?
3. Will the use of a Healthcare passport help overcome problems of communication between people with dementia and health and care services?
4. Will a Healthcare passport empower and support the autonomy and decision-making of people with dementia and family carers?
5. What is the level and quality of engagement with such a passport by health and social care professionals?
6. How do the needs of people with dementia and their family carers, with regard to service contacts and communication, evolve over time?

## Methods/design

We will undertake a realist review of the use of communication tools for people living with dementia followed by a qualitative longitudinal study (QLR) in order to record the experiences of users of the healthcare passport over time.

**A realist review**: We will use a realist review of the literature on communication tools in dementia [19, 20]. The realist review is a relatively new but rigorously inclusive method of examining the mechanisms, often hidden, that effect positive (or negative) outcomes in a program. Rather than looking simply at whether or not an intervention works, a realist review seeks to elucidate “what works for whom in what circumstances and why?” Of considerable benefit to our medium term goal of design and implementation, this approach will inform the theoretical linkage between communication and decision-making in dementia care and their relationship with personhood. It will also provide a framework for a better explication of these as complex and evolving phenomena, determined by a constellation of structural and cultural factors and processes [21]. A joint working group of the academics, clinicians and family carers will undertake a scope of the review, in which we will delineate the review purpose, key question(s), and underlying theory(ies). The findings from the review, incorporating both quantitative and qualitative studies [22] will inform the development of a framework for the evaluation of the healthcare passport and will support the production of more general recommendations for policy and practice in dementia care.

**Qualitative Longitudinal Research (QLR)** uses mainly in-depth interviews and is invaluable when recording and exploring change over time, and their associated processes [18]. Although relatively time consuming than one-off interviews in which a person is invited to reflect on past events, QLR can help explore current situations and anticipated issues and then also seek to understand how these unfold in ways that are planned or not planned [23]. In this way, we can investigate, back and forth, through the concerns and experiences of the participants as they respond to emerging events and needs. Data will be collected through the following methods: interviews with people with dementia and family caregivers; online questionnaires and brief follow-up phone interviews with health and social care professionals; Analysis of the content of the passport. A research management board has been established to oversee the study, and will include regular contact with a service user engagement group through the Alzheimer’s Society throughout.

### Ethics and informed consent

There are a number of key ethical issues related to this study. People with dementia have varying degrees of capacity and this is likely to change over time due to dementia and a range of psychosocial, situational, medical, psychiatric and neurological factors. A threshold for competence relates to individual tasks including the competence to make a decision about participating in research. The importance of making such a distinction is to determine whether the potential participant should consent to participating in a particular study or whether somebody else should make that decision on his/her behalf. While there are some difficulties in conducting research involving those who cannot fully consent, there are also ethical issues associated with excluding these individuals from research, and thus denying the potential benefits of the study. The study has been awarded ethical approval from the Office of Research Ethics Committee Northern Ireland.

### Sample selection and recruitment

The project will be undertaken in collaboration with the Alzheimer’s Society (AS) and a lead clinician from local memory clinics, who will oversee the recruitment of service users and the delivery of the healthcare passport. Potential participants will be selected randomly from lists of patients in memory clinics in a Health and Social Care Trust in Northern Ireland. Only the lead clinician will have access to patient data. Given the frequency of non-participation and drop-out rates in studies of this type, data will be collected on reasons for declining to participate and/or leaving the study. We will recruit 20-25 patients, and include their family carers in the study as part of a care dyad. The sample is relatively large for a qualitative study but this is intended to assist maximum variation (e.g. age, gender, social class, support needs, co-morbidity) and to help offset attrition to the study.

A list of key health and care staff will be obtained from the patient and caregiver, and consent to contact them. We will seek to confirm capacity to participate in this study with responsible clinicians and will continue to check with families and professionals on the mental health status of the person with dementia and capacity issues related to consent and continued involvement in the study. Where individuals are deemed to be unable to consent once the study has begun, we have established a procedure to obtain proxy consent, in accordance with NIHR GCP guidelines and the guidance on ethics of dementia research from the Nuffield Foundation. Following this, we will contact GPs and follow up with a phone call. We will meet with the GP to explain the use of the tool, leave explanatory written information and details of how to access the film for all interested staff, and access a list of healthcare professionals who are involved in care of the individual. These staff will also need to be contacted and given information on when and how to use the Passport, by telephone discussion, via the web based film and via emailed written information on the passport and its use (http://www.rcgp.org.uk/rcgp-near-you/rcgp-northern-ireland/my-healthcare-passport.aspx). The research will be based in a relatively small area, allowing us to offer staff groups training on the purpose and use of the passport.

### Preparation for passport use

A healthcare professional will provide training to an Engagement and Participation Officer (EPO), based wit the Alzheimer’s Society, who will support and advise participants on the passport’s use. Following consent, the EPO will arrange an appointment to meet individual participants and a family member. Although the capacity of the person with dementia may diminish over the evaluation period, we intend that they are fully involved in using the passport, and any associated decision-making. Throughout the study, the EPO will record capacity and involvement issues. Individuals who agree to take part will be given a one to one information session (approx. 1 h) on the purpose and use of the Passport and will be invited to bring a support person. We will explain the purpose of using the tool and how to complete it. This will also be provided in writing, so it can be shared with others.

### Interviews with service users and carers

Following written informed consent, the interviews will be undertaken soon after training, then approximately 3 and 6 months later. Structured topic guides for each of the three interview stages will cover the main research questions. Briefly, the first interview will cover personal and social contexts, health and social needs (difficulties, assets, coping strategies), and current health & social care service use. Subsequent interviews will cover the use of the passport.

### Analysis

The interviews will be audiotaped, transcribed and then entered into a qualitative software program (N-Vivo version 9) for data coding and management. The data analysis will be informed by the findings from the realist review which will be incorporated into a Framework approach [24, 25]. Thus, we will code and index the data, using a spreadsheet in order to generate a matrix into which the data will be ‘charted’. This involves summarizing the data by category from each transcript, building themes with the support of memos and data display (matrices and diagrams). Transcripts will be analysed and coded independently. Some specific areas that we will cover: (a) a retrospective examination of the experience of people with dementia, family carers, help-seeking and communication needs – prior to using the passport; (b) practical use of the passport, differentiated by different care characteristics and contexts (e.g. dementia stage, social class and social support networks, gender and care-relationship); (c) change in use of the passport over time in response to need; (d) care planning and advance directives for end of life care; (e) joint decision making (family and patient); (f) comprehension and ease of use by stakeholders (professionals and family); and (g) reasons for discontinuation.

From our realist systematic review, we will seek to develop specific questions on contextual and intervening conditions that may additionally help guide analysis. The differential quality and quantity of information that is shared by families and professional services – for example, what type of information is withheld, if any, by whom and for what reasons? What might be the unintended consequences of using the passport? Where appropriate, we will adopt a narrative approach in order to illustrate the complex issues that arise. Thus, we will attempt to combine a deeper understanding and portrayal of the experiences of the participants while also situating and interpreting these experiences within their wider personal (and medical) contexts and with reference to specific events of significance to the person with dementia and family members. Using this approach we will attempt to understand the commonalities while exploring and highlighting issues that arise from unique or different perspectives; family support or complexity of care, for example. Particular note will be taken of new themes arising and any unusual perspectives. In order to maximise the usability and reliability of the findings, we will use the Consolidated Criteria for Reporting Qualitative research (COREQ) checklist in our writing up. (http://www.equator-network.org/reporting-guidelines/coreq/)

### Passport content analysis

The passports’ contents are expected to be a rich source of data about the things that matter to patients and families and about their pathways and experiences within health and social care services. We will undertake a content analysis of the passports, providing a detailed report on how they are used and by whom, in addition to family carers, and the level and quality of the information provided. Again, we will seek in the passport for person-centred care such as evidence of: maintaining social connections; the person’s context being accounted for in plans; meaningful relationships being nurtured; evidence of a person’s values being considered. This will include an examination of any relevant information that appears to be missing. Other data: we will provide detailed information about (1) the uptake of the passport; (2) an estimate of trial recruitment; (3) reasons for family engagement and sustainability of passport use; (4) engagement with health and social care professionals; (5) training and support issues.

### Dissemination

The applicants and collaborating organisations will provide study progress and findings through social media outlets, including linkage to conference events, reports, materials and publications. An end of study report will be placed on the website and be disseminated through the CRN-MH and the dementia networks in the UK. Findings will be published in high quality, open access journals.

### Knowledge transfer

The study will generate important knowledge on dementia service users’ and family caregivers’ service experiences, communication and decision-making, much of which will contribute to education and training across a range of clinical and community based services. Findings will be of considerable interest and benefit to a range of health and care organizations beyond that of the immediate community of interest (dementia). Thus, there are knowledge transfer implications in developing services and interventions for a range of service users and family caregivers more generally including mental health and those managing life-limiting conditions and end of life care.

## Discussion

A shift away from the medical model of health and social care provision has led to increasing attention to more democratised care, service user engagement and patient-led services. However in the case of people living with dementia, particularly those with complex healthcare needs, there has been less progress in improving continuity of care and a person centred approach. Furthermore, family carers are often left to manage the gaps in service provision, and join the dots between care providers. There is evidence that tools that emphasise effective communication between patients’ care dyads and healthcare professionals can increase the likelihood of more joined-up and compassionate care. The Healthcare Passport was designed to encourage self-management and control of one’s own personal information to preserve or enhance self-worth, agency, social confidence and a sense of security amidst change to improve quality of life throughout a complex treatment journey, and to convey the importance of the whole individual to the health and social care providers along the way. There are, however, many potential variables in the success or failure of such an intervention. Unintended consequences, including the creation of unrealistic expectations on all sides, decreasing direct person-to-person communication by emphasising the use of a written tool, must be investigated. Consideration must also be given to the context of the intervention, and assessing in what circumstances and for whom the passport is less or more effective. This multi-modal methodology allows for the collection of data from a number of perspectives, in a range of ways, and over a period of time, thus providing a strong evidence base on both the feasibility of the application of the Passport, and an assessment of how and why it does or does not improve communication and quality of life for users. The realist review and participation of service users in the overall management of the study will allow for innovative and inclusive theoretical developments and data analysis, and has the potential to highlight practical methodological best practice and pitfalls in working with participants with complex needs and fluctuating capacity and in the recruitment and retention of family carers in dementia research.

## Abbreviations

AS, Alzheimer’s Society; EPO, Engagement and Participation Officer
